# Quantum Relative Entropy of Tagging and Thermodynamics

**DOI:** 10.3390/e22020138

**Published:** 2020-01-24

**Authors:** Jose Diazdelacruz

**Affiliations:** Department of Applied Physics and Materials Engineering, Universidad Politecnica de Madrid, 28040 Madrid, Spain; jose.diazdelacruz@upm.es

**Keywords:** information heat engines, quantum thermodynamics, quantum relative entropy

## Abstract

Thermodynamics establishes a relation between the work that can be obtained in a transformation of a physical system and its relative entropy with respect to the equilibrium state. It also describes how the bits of an informational reservoir can be traded for work using Heat Engines. Therefore, an indirect relation between the relative entropy and the informational bits is implied. From a different perspective, we define procedures to store information about the state of a physical system into a sequence of *tagging qubits*. Our labeling operations provide reversible ways of trading the relative entropy gained from the observation of a physical system for adequately initialized qubits, which are used to hold that information. After taking into account all the qubits involved, we reproduce the relations mentioned above between relative entropies of physical systems and the bits of information reservoirs. Some of them hold only under a restricted class of coding bases. The reason for it is that quantum states do not necessarily commute. However, we prove that it is always possible to find a basis (equivalent to the total angular momentum one) for which Thermodynamics and our labeling system yield the same relation.

## 1. Introduction

The role of information in Thermodynamics [1,2] already has a long history. Arguably, it is best exhibited in Information Heat Engines [3,4,5,6,7,8,9,10,11,12,13,14,15,16,17,18,19,20,21,22]. They are devices that cyclically extract energy from a thermal reservoir and deliver it as mechanical work. They do so by increasing the entropy of a set of bits from an *information reservoir* [1,23,24,25,26,27,28,29,30]. There are differences between classical bits and quantum qubits [31,32,33,34,35,36], but they share the same maximum efficiency [37,38]. Appendix A describes a basic model of an Information Heat Engine.

Physical systems in a state ρ out of thermal equilibrium also allow the production of work. It turns out to be related to the relative entropy $S\left(\rho ||\tau \right):=\mathrm{Tr}\;\left\{\rho \log\rho -\rho \log\tau \right\}$ with respect to the equilibrium state τ, again an informational quantity (in this paper, log(x) always represents the binary logarithm of *x*). Appendix B contains a short derivation of this result. Some recent reviews compile a variety of properties and functional descriptions of relative entropy [39,40,41,42]. Probably, the most closely connected to this paper is its interpretation as the average extra number of bits that are employed when a code optimized for a given probabilistic distribution of words is used for some other. This paper contributes a new procedure that also reveals a direct connection between the relative entropy of physical systems and information reservoirs circumventing Thermodynamics. It focuses on the quantum case, particularly when the relative entropy is defined for non-commuting density matrices.

**Figure 1:**
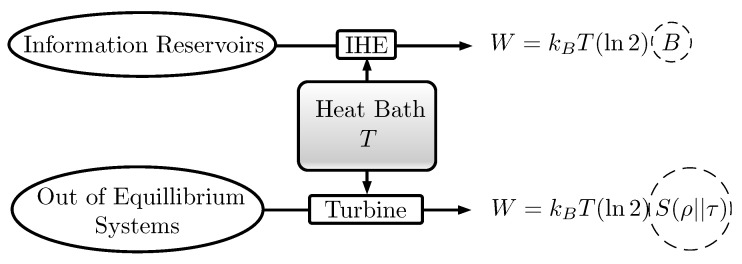
According to Thermodynamics, work can be reversibly obtained from a heat bath by consuming *B* bits of an information reservoir and also by decreasing its relative entropy S(ρ||τ) with respect to the thermal state τ.

The generation of work in Information Heat Engines always requires the transfer of thermal energy from a heat reservoir and needs adequate steering of a Hamiltonian. In Szilard Engines [14,43,44], they occur at the same time as the piston moves within the cylinder. In the one particle case, every bit from an information reservoir enables the generation of $k_{B}\left(\ln2\right)T$ mechanical work. Other thermal machines, such as turbines, also imply tuning Hamiltonians and heat transfer. Combining these devices, it is possible to produce work by increasing the entropy of informational qubits and use it to build up the relative entropy of a physical system with respect to its thermal equilibrium state. The net heat and work would vanish (see Figure 1). This consideration naturally motivates the question of whether it would be possible to do the same transformation only with *informational* manipulations, without any reference to Hamiltonians, temperatures or heat baths.

In order to simplify the quantification of the resources involved, we exclusively consider unitary operations. In addition, we only allow observational interactions on the physical system. This restricts the set of transformations to those defined by controlled unitary gates, in which the state of the physical system remains in the controlling part. Basically, we compute the informational cost of *labeling* a physical system by considering the number of pure $|0\rangle$ state tagging qubits at the input minus those recovered at the output. In the following, we may use the terms “initialize” or “reset” to denote the action of driving a tagging qubit to a $|0\rangle$ state.

The tagging operation implies using some coding procedure to correlate the quantum states of a physical system and its label. Conversely, we also consider the process of deleting that correlation and returning the tagging qubits to an information reservoir. We assume that the code has to be optimal in the sense that it uses the least average amount of bits to identify the state of a physical system. Averaging is defined with respect to an underlying probability distribution of states. For this reason, we choose a Shannon coding technique; it is asymptotically optimal and provides a simple relation between the lengths and the probabilities of the codewords. We consider two degrees of tagging. The *tight-labeling* implies a reversible assignation of a label to every physical system. It is described in Section 2. The *loose-labeling* implies *tight-labeling* a collection of physical systems followed by a random *shuffling*. It is studied in Section 3. The discussion is presented in Section 4 and the conclusions in Section 5. A simple example, using magnetic spins, is given in Appendix E, in order to illustrate some of the ideas presented in the paper.

## 2. Tight Labeling

In this section, we present a method for writing a label, consisting of a set of qubits, with the purpose of representing the quantum state of a physical system. We call it *tight* because each label is assigned unambiguously to the state of the physical system that it is attached to.

**Figure 2:**
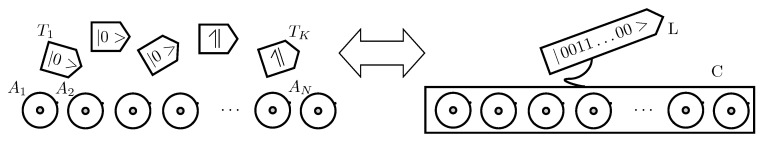
Our labeling procedures assume the availability of tagging qubits in either a $|0\rangle$ or a maximally mixed state, and physical systems, referred to as *atoms*. The labeling assigns a set of *H* tagging qubits to a cluster of *N* atoms. The cost is defined as the number of tagging qubits in state $|0\rangle$ employed.

We consider a sufficiently large set of identical physical systems that will be referred to as *atoms*. For simplicity, a two-dimensional Hilbert space for them is assumed. The statistical distribution for each atom is determined by its quantum state, which is known to be σ. The eigenstates of σ are denoted by $|↑\rangle ,|↓\rangle$ and their eigenvalues are ordered as $\sigma_{↓}\leq \sigma_{↑}$. Atoms are grouped in *clusters* of a common length *N*. Besides the atoms, we assume unlimited availability of *tagging qubits* in either a pure $|0\rangle$ or a maximally mixed state (see Figure 2).

We consider a *coding basis* $B_{C}$ that diagonalizes $\sigma^{⊗N}$. Its $2^{N}$ vectors are denoted by ${|b_{1}\ldots b_{N}\rangle }_{B_{C}}$, where $b_{i}$ can be either 0 or 1.

The operations considered are:

1. Any unitary transformation on a system *T* of tagging qubits.
2. Unitary transformations on joint states of cluster *C* and a system *T* of *K* tagging qubits, U:(C⊗T)→(C⊗T) that are defined, using the $B_{C}$ basis for *C* and the computational basis $B_{T}$ for *T*, by:
  (1) $$U_{f}\;{|b_{1}\ldots b_{N}\rangle }_{B_{C}}⊗|t_{1}\ldots t_{K}\rangle ={|b_{1}\ldots b_{N}\rangle }_{B_{C}}⊗{|\left(t_{1}\ldots t_{K}\right)⊕f\left(b_{1}\ldots b_{N}\right))\rangle }_{B_{T}},$$
  where $f(b_{1}\ldots b_{N})$ is any function that transforms binary strings of *N* bits into strings of *K* bits. They can be considered as just probing operations with respect to clusters. It is also easy to check that $U_{f}=U_{f}^{†}$.

The *cost* of any operation will be tallied as the number of tagging qubits in a pure $|0\rangle$ state that are input and not recovered at the output.

We further choose a binary Shannon lossless code for the $b_{1}\ldots b_{N}$ sequences according to a frequency given by the eigenvalue $\sigma_{b_{1}\ldots b_{N}}$ of $\sigma^{⊗N}$ that corresponds to ${|b_{1}\ldots b_{N}\rangle }_{B_{C}}$. The procedure defines a function $c(b_{1}\ldots b_{N})$ that assigns a binary codeword to every string $b_{1}\ldots b_{N}$. Let *H* be the maximum length of all the codewords. We further define a *label* as an array of *H* tagging qubits. The $2^{H}$ vectors of the label computational basis $B_{L}$ are denoted by ${|\ell_{1}\ldots \ell_{H}\rangle }_{B_{L}}$, where $\ell_{i}$ can be either 0 or 1.

The coding procedure determines a unitary operator $U_{c}$ that acts on every cluster–label pair (C,L). It can be defined by specifying how the elements of the basis $B_{C}⊗B_{L}$ transform; it is given by:

(2) $${|b_{1}\ldots b_{N}\rangle }_{B_{c}}⊗{|\ell_{1}\ldots \ell_{H}\rangle }_{B_{L}}\to {|b_{1}\ldots b_{N}\rangle }_{B_{c}}⊗{|c^{*}\left(b_{1}\ldots b_{N}\right)⊕\left(\ell_{1}\ldots \ell_{H}\right)\rangle }_{B_{L}},$$

where $c^{*}\left(b_{1},\ldots ,b_{N}\right)$ represents the Shannon codeword $c(b_{1},\ldots ,b_{N})$ supplemented with the necessary trailing 0s to match a length *H*.

**Figure 3:**
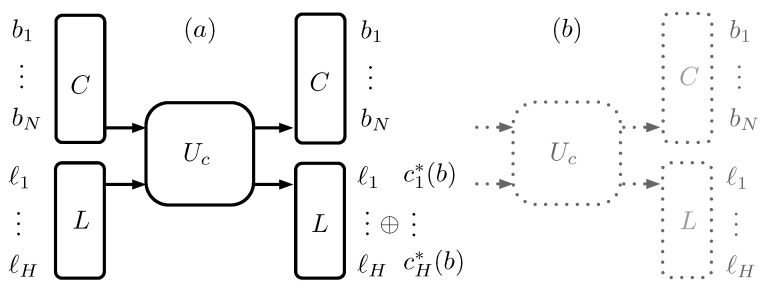
(**a**) represents the coding procedure as a unitary operation $U_{c}$, controlled by the cluster *C*, on the tagging qubits of the label *L*. If the tagging qubits are initially in a $|0\rangle$ state, they hold the coded string for the cluster; (**b**) represents the inverse operation, which is equal to $U_{c}$, so that $U_{c}^{2}$ is the identity transformation.

Labeling an *N*-atoms cluster *C* in state ρ means applying the unitary operator $U_{c}$ to the cluster and a label of *H* tagging qubits in a ${|0\ldots 0\rangle }_{B_{L}}$ state, as is depicted in Figure 3. When ρ is diagonal in the $B_{C}$ basis, the operation is equivalent to the classical operation of coding according to the Shannon method and storing the codeword in the label. The resulting state is a classical statistical mixture of cluster–label pairs.

**Figure 4:**
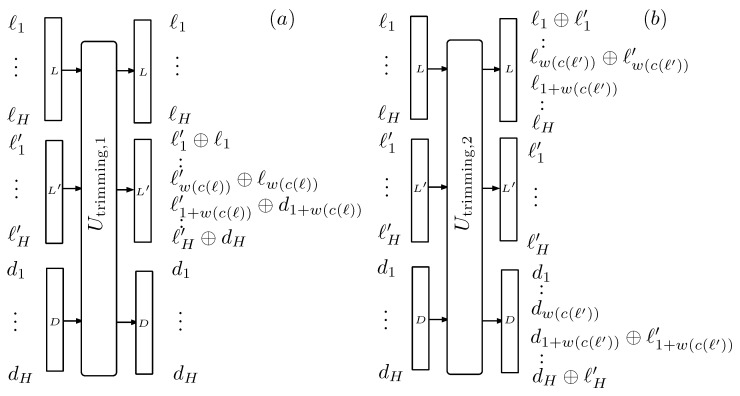
Representation of the procedure employed for replacing the trailing qubits that need not be used in the codeword by maximally mixed ones, as explained in Appendix C. It is split into two unitary transformations. The first one, represented in (**a**), copies the first w(c(ℓ)) qubits of *L* into $L^{\prime }$ that enters with all its tagging qubits in the $|0\rangle$ state. The remaining qubits are copied from the maximally mixed qubits of *D*. The second transformation, represented in (**b**), resets the *L* label and the $H-w(c\left(\ell^{\prime }\right))$ trailing qubits of *D*. The overall function is recovering $H-w(c\left(\ell^{\prime }\right))$ qubits in state $|0\rangle$ and generate a new label with maximally mixed trailing qubits.

We define the width $w(c\left(b_{1}\ldots b_{N}\right))$ of the codeword $c(b_{1}\ldots b_{N})$ assigned to the ket ${|b_{1}\ldots b_{N}\rangle }_{B_{C}}$ as the number of bits in $c(b_{1}\ldots b_{N})$. Only the leading $w(c\left(b_{1}\ldots b_{N}\right))$ qubits of the label contain information. The $H-w(c\left(b_{1}\ldots b_{N}\right))$ trailing qubits of *L* are superfluous and should be replaced by others in a completely mixed state. For this purpose, we define a new unitary operation $U_{\mathrm{trimming}}$ by which the trailing qubits are swapped with those of a new label *D*. In the labeling process, *D* contains *H* maximally mixed qubits. The operation of $U_{\mathrm{trimming}}$ is depicted in Figure 4 and explained more elaborately in Appendix C.

In our setting, the width of the codeword is a quantum variable represented by the operator $\hat{W}$, which operates on the Hilbert space of the cluster. Its eigenvectors are those of the $B_{C}$ basis, and its eigenvalues are the widths of their Shannon codewords. Its effect on the basis vectors is:

(3) $${|b_{1},\ldots ,b_{N}\rangle }_{B_{c}}\to w\left(c\left(b_{1},\ldots ,b_{N}\right)\right){|b_{1},\ldots ,b_{N}\rangle }_{B_{c}}$$

and also represents the *cost* of the labeling procedure. We further define the *atomic* width ${\hat{W}}_{1}$ as $\hat{W}/N$. For sufficiently large *N*, $w(c\left(b_{1},\ldots ,b_{N}\right))/N$ converges to $-\log\sigma_{b_{1},\ldots ,b_{N}}/N$. Accordingly,

(4) $${\hat{W}}_{1}\to -\left(\log\sigma^{⊗N}\right)/N.$$

Thus, for sufficiently large *N*, the average atomic width $W_{1}\left(\rho \right)$ for a codeword in state $\rho^{⊗N}$ is given by:

(5) $$W_{1}\left(\rho^{⊗N}\right)=-\frac{1}{N}\;\mathrm{Tr}\;\left\{\rho^{⊗N}\log\sigma^{⊗N}\right\}=-\frac{1}{N}\;\left(S\left(\rho^{⊗N}\right)+S(\rho^{⊗N}\left|\right|\sigma^{⊗N})\right),$$

which, taking into account that $S\left(\rho^{⊗N}\right)=NS\left(\rho \right),\;S(\rho^{⊗N}\left|\right|\sigma^{⊗N})=NS(\rho ||\sigma )$, can be rewritten as

(6) $$W_{1}\left(\rho^{⊗N}\right)=S\left(\rho \right)+S\left(\rho ||\sigma \right)$$

and represents the *atomic cost* of labeling clusters in state $\rho^{⊗N}$. It is straightforward to reverse the process and check that an atomic yield of

(7) $$Y_{1}\left(\rho^{⊗N}\right)=S\left(\rho \right)+S\left(\rho ||\sigma \right)$$

is obtained.

A cluster in state $\rho^{⊗N}$ can be described as being in a probabilistic mixture of eigenstates of $\rho^{⊗N}$. Each of them defines a tight-label that allows for unambiguously identifying which eigenstate of $\rho^{⊗N}$ it is attached to. The cost expressed by Equation (6) represents the average length of the codewords that correspond to clusters drawn according to the distribution defined by $\rho^{⊗N}$. The equivalent situation in Thermodynamics corresponds to averaging the work that is necessary to produce pure state physical systems (as spin systems in the example of Appendix E) out of equilibrium, following the distribution given by $\rho^{⊗N}$. However, there is a subtle difference from the case that we want to model in the next section. In it, we still have clusters whose states are drawn from the distribution defined by $\rho^{⊗N}$, but we ignore the particular state of each cluster. To cope with this new situation, it would be sufficient to overlook the precise label that is attached to each cluster, but keeping the distribution that corresponds to $\rho^{⊗N}$. This is implemented through a process of label shuffling for a collection of clusters in state $\rho^{⊗N}$. The procedure is described in Section 3.

## 3. Loose Labeling

**Figure 5:**
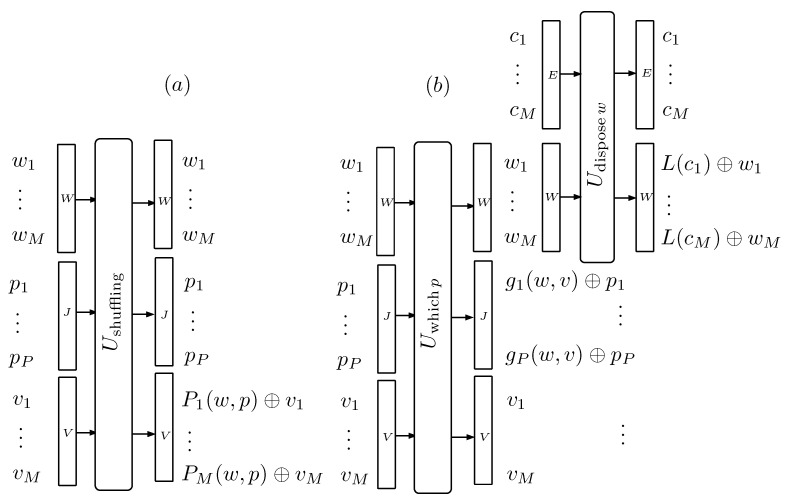
The figure describes the unitary process used to shuffle the labels $w_{1},\ldots ,w_{M}$ of a collection *F* of *M* tight-labeled clusters, as is explained in Appendix D. Besides the labels, a set of *P* maximally mixed qubits $p_{1},\ldots ,p_{P}$ and a set of *M* labels $v_{1},\ldots ,v_{M}$, all in state ${|0\ldots 0\rangle }_{B_{L}}$ enter the first unitary gate (**a**). It shuffles the labels $w_{1},\ldots ,w_{M}$, according to the permutation indicated by the $p_{1},\ldots ,p_{P}$ qubits and stores the result in the $v_{1},\ldots ,v_{M}$ labels. The second gate (**b**) resets the qubits that signaled the permutation. The last gate resets the $w_{1}\;\ldots ,w_{M}$ labels by regenerating them from the $c_{1},\ldots ,c_{M}$ clusters.

The tagging procedure put forth in the previous section outputs maximally correlated cluster-label pairs. In this section, we describe a procedure that reduces the correlation. To this end, the label *L* assigned to cluster *C* will be the codeword of another cluster $C^{\prime }$ that belongs to a collection *F* of *M* clusters, all of them in state $\rho_{C}=\rho^{⊗N}$. Therefore, the state of *F* is $\rho_{F}={\left(\rho^{⊗N}\right)}^{⊗M}=\rho^{⊗NM}$. Figure 5 represents the process with unitary gates that acts on a collection of *M* labeled cluster pairs $w_{1}-c_{1},\ldots ,w_{M}-c_{M}$, a random set of *P* tagging qubits $p_{1},\ldots ,p_{P}$ and an auxiliary label collection $v_{1},\ldots ,v_{M}$. The role played by $p_{1},\ldots ,p_{P}$ is to generate a random shuffling of the labels. The process is analyzed in Appendix D, where it is shown that the average number R(F) of qubits in the $p_{1},\;\ldots ,p_{P}$ array that exit in a $|0\rangle$ state verifies that, for large *M*, $\frac{R(F)}{M}$ converges to $S_{L}$, where $S_{L}$ is the entropy associated with the probability distribution for the $2^{N}$ possible labels of the $2^{N}$ elements of the $B_{C}$ basis.

Therefore, taking into account the cost of tight-labeling the *F* collection, given by Equation (5), the one of loosely tagging the clusters of *F* is

(8) $$W^{\left(L\right)}\left(F\right)=M\left(S\left(\rho^{⊗N}\right)+S(\rho^{⊗N}\left|\right|\sigma^{⊗N})-S_{L}\right),$$

which leads to a value per atom:

(9) $$W_{1}^{\left(L\right)}=\frac{1}{N}\left(S\left(\rho^{⊗N}\right)-S_{L}+S(\rho^{⊗N}\left|\right|\sigma^{⊗N})\right).$$

Next, we will find a suitable expression for $S_{L}$. Each label $c(b_{1}\ldots b_{N})$ is assigned to a vector ${|b_{1}\ldots b_{N}\rangle }_{B_{C}}$ of the $B_{C}$ basis. Its frequency is given by $\langle b_{1}\ldots b_{N}|\rho^{⊗N}|b_{1}\ldots b_{N}\rangle$. The probabilities for the set of codewords are the eigenvalues of the density matrix

(10) $${E\left(\rho^{⊗N}\right)}_{B_{C}}:=\sum_{b_{1},\ldots ,b_{N}}|b_{1}\ldots b_{N}\rangle \langle b_{1}\ldots b_{N}|\rho^{⊗N}|b_{1}\ldots b_{N}\rangle \langle b_{1}\ldots b_{N}|.$$

It is immediate to check that $S_{L}=S\left({E\left(\rho^{⊗N}\right)}_{B_{C}}\right)$. Thus, Equation (9) can be rewritten as

(11) $$W_{1}^{\left(L\right)}=\frac{1}{N}\;\left(S\left(\rho^{⊗N}\right)-S\left({E\left(\rho^{⊗N}\right)}_{B_{C}}\right)+S(\rho^{⊗N}\left|\right|\sigma^{⊗N})\right).$$

Notice that ${E\left(\cdot \right)}_{B_{C}}$ represents a CPTP (Completely Positive Trace Preserving) map, which is not a unitary transformation. However, all the operations of our labeling system are unitary. The CPTP map is used here just as a means to find a convenient expression for $S_{L}$, not as a real operation on the state of the clusters and labels.

Next, we define a particular base $B_{C}$ for which Equation (11) will only retain the last term in the N→∞ limit.

Let X,Y,Z be the operators represented by the Pauli matrices in the $|↑\rangle ,|↓\rangle$ base, which diagonalizes σ. In the Hilbert space of the i-th atom of a cluster, they are denoted by $X_{i},Y_{i},Z_{i}$. For the whole cluster, we define:

(12) $$S_{x}:=\sum_{i=1}^{N}X_{i},\;S_{y}:=\sum_{i=1}^{N}Y_{i},\;S_{z}:=\sum_{i=1}^{N}Z_{i},\;S^{2}=S_{x}^{2}+S_{y}^{2}+S_{z}^{2}.$$

Let $B_{M}$ be the basis which diagonalizes $S_{z},S^{2}$ (the well-known momentum basis in Quantum Physics).

In the $B_{M}$ basis, any state $\rho^{⊗N}$ is a mixture of states $\rho^{⊗N}=\sum r_{i}\rho_{i}$, where $\rho_{i}$ is a state defined within the i−th invariant subspace of $S^{2}$. The entropy of this mixture is the sum of the entropy $S_{r}$ associated with the mixture and the weighted entropy of the different $\rho_{i}$:

(13) $$S\left(\rho^{⊗N}\right)=-\sum_{i}r_{i}\log r_{i}+\sum_{i}r_{i}S\left(\rho_{i}\right).$$

The same decomposition can be applied to the ${E\left(\rho^{⊗N}\right)}_{B_{M}}$ state. Taking into account that $\rho^{⊗N},S^{2}$ commute,

(14) $${E\left(\rho^{⊗N}\right)}_{B_{M}}=\sum_{i}r_{i}{E\left(\rho_{i}^{⊗N}\right)}_{B_{M}},$$

so that using Equation (13) for $S({E\left(\rho^{⊗N}\right)}_{B_{M}})$, and substracting both entropies, we arrive at

(15) $$S\left(\rho^{⊗N}\right)-S\left({E\left(\rho^{⊗N}\right)}_{B_{M}}\right)=\sum_{i}r_{i}\left(S\left(\rho_{i}\right)-S\left({E\left(\rho_{i}^{⊗N}\right)}_{B_{M}}\right)\right).$$

The maximum entropy of a state $\rho_{i}$ is $\log d_{i}$, where $d_{i}$ is the dimension of the i-th invariant subspace. Its maximum value is N+1. Therefore, the absolute value of the right half side of Equation (15) can not be greater than log(N+1). In the N→∞ limit,

(16) $$\lim_{N\to \infty }\frac{S\left(\rho^{⊗N}\right)-S\left({E\left(\rho^{⊗N}\right)}_{B_{M}}\right)}{N}=0,$$

so that, for sufficiently large *N*,

(17) $$W_{1}^{\left(L\right)}=S\left(\rho ||\sigma \right).$$

## 4. Discussion

A well-known situation in Thermodynamics is the availability of a thermal reservoir of freely available, non-interacting atoms in a Gibbs state, given by:

(18) $$\tau =Z^{-1}\left(\hat{H},T\right)\;e^{-\frac{\hat{H}}{k_{B}\;T}},\;Z\left(\hat{H},T\right)=\mathrm{Tr}\left\{e^{-\frac{\hat{H}}{k_{B}\;T}}\right\},$$

where $\hat{H}$ is the Hamiltonian of each atom, *T* is the temperature and $Z(\hat{H},T)$ represents the partition function.

A cluster of atoms in another state ρ can be used to obtain work from the energy of the thermal reservoir. The obtainable work per atom, as given in [41,45] and derived in Appendix B, is

(19) $$W_{\rho \to \tau }=k_{B}\left(\ln2\right)T\;S\left(\rho ||\tau \right)$$

and is usually collected by some *physical* means (mechanical, electromagnetic, etc.) which involves coupling to thermal baths and mechanical or electromagnetic energy-storage systems. Remarkably, it only depends on the relative entropy S(ρ||τ) that is connected to the physics through the dependence of τ on the Hamiltonian $\hat{H}$ and the temperature *T*. The work obtained matches the heat transferred from the thermal reservoir plus the decrease of the internal energy of the atom. After the process, the state of the atom is τ. The reverse operation, driving a system initially in state τ to an out of equilibrium state ρ demands the same amount of work to be supplied in the process.

In this paper, we have come across the relative entropy from a very different approach. We have chosen to employ a coding system that asymptotically minimizes the labeling cost for τ. Shannon coding for state τ satisfies this requirement. In the process of labeling, we incur a cost that can be evaluated after substitution of τ for σ in Equation (17), in the case of loose-labeling. It is equivalent to the process of driving a system from state τ to state ρ, with the following observation: while in the Thermodynamic operation the process of transforming requires work, in the labeling approach, it needs qubits in the $|0\rangle$ state.

In a different Thermodynamic setting, we know that work can also be obtained from informational qubits at Information Heat Engines in the presence of a heat bath. Typically, they enter in a known pure state (say $|0\rangle$) and exit in a maximally mixed one. They need to be coupled to a physical system that should be able to equilibrate with a thermal reservoir and couple to an external energy storage. The work obtained per qubit is

(20) $$W_{\mathrm{IHE}}=k_{B}\left(\ln2\right)T.$$

Accordingly, it is clear that the relative entropy of physical systems with respect to a thermal state can be traded for bits of information reservoirs by means of engines and heat baths within a Thermodynamic context.

We claim that, in this paper, we have described a way to do the same with purely informational manipulations. Furthermore, the physical system is accessed for probing operations that reduce to acting on the information qubits according to its state. Labeling is a particular kind of these processes.

However, the loose-labeling cost, given by Equation (11), depends on the labeling strategy through the choice of the coding basis $B_{C}$. The atomic cost does not converge to the relative entropy unless $S(\rho^{⊗N})$ converges to $S({E\left(\rho^{⊗N}\right)}_{B_{C}})$. This is trivial if ρ,σ commute, but, in a general quantum scenario, it can not be assumed. Nonetheless, even in the non-commuting case, it is accomplished by using the eigenbasis described in Section 3. For the general case, when another basis is chosen, $S(\rho^{⊗N})$ does not converge to $S({E\left(\rho^{⊗N}\right)}_{B_{C}})$ and the loose-labeling costs are lower than the quantum relative entropy. This can be deduced by substracting both:

(21) $$S\left({E\left(\rho^{⊗N}\right)}_{B_{C}}\right)-S\left(\rho^{⊗N}\right)=S(\rho^{⊗N}\left|\right|\sigma^{⊗N})-S({E\left(\rho^{⊗N}\right)}_{B_{C}}\left|\right|\sigma^{⊗N}),$$

where we have taken into account that, because $\sigma^{⊗N}$ is diagonal in $B_{C}$, then

(22) $$\mathrm{Tr}\;\left\{{E\left(\rho^{⊗N}\right)}_{B_{C}}\;\log\sigma^{⊗N}\right\}=\mathrm{Tr}\;\left\{\rho^{⊗N}\;\log\sigma^{⊗N}\right\}.$$

Notice that $\sigma^{⊗N}={E\left(\sigma^{⊗N}\right)}_{B_{C}}$, so that Equation (21) can be written as

(23) $$S\left({E\left(\rho^{⊗N}\right)}_{B_{C}}\right)-S\left(\rho^{⊗N}\right)=S(\rho^{⊗N}\left|\right|\sigma^{⊗N})-S({E\left(\rho^{⊗N}\right)}_{B_{C}}\left|\right|{E\left(\sigma^{⊗N}\right)}_{B_{C}}),$$

which is always positive by the monotonicity of quantum relative entropy.

At any point, tracing out the label places the reduced state of the cluster back to σ. Therefore, we interact with the cluster just to obtain or delete information about it. The parallel with the situation in Thermodynamics is clear: bits from information reservoirs are traded for changing the relative entropy of state ρ with respect to σ,τ. From this point of view, the most important aspect of a physical system in a thermodynamical setting is knowing its state, so that it can be used to supply the corresponding work. It is the state that fixes the process by which work is obtained.

The two types of labeling described exhibit different costs. It is quite obvious because the loose-labeling implies shuffling. This leads to labels that are related not to a particular cluster, but to a collection of them that share some particular state. It is natural that the work given by Equation (19) is related to this cost because it assumes a process which is common to all of them. However, if we have a tight-labeled collection of clusters, we can process each one in a different way, chosen according to its label. Then, each cluster would contribute a work given by the relative entropy of the pure state ${|b_{1}\ldots b_{N}\rangle }_{B_{C}}$ identified according to the label. The average work value would be given by:

(24) $$<W>=k_{B}\left(\ln2\right)T\;\left(S(\rho )+S(\rho ||\tau )\right),$$

which corresponds to the cost of tight-labeling, given by Equation (6), irrespective of the particular choice of the coding basis.

From another perspective, loose-labeling is essentially the process of disarranging the tight-labels of a collection of *M* clusters. Let us first assume that σ,ρ commute. Asymptotically, as M→∞, the number of possible orders for the set of labels tends to MS(ρ), which is precisely the difference between Equations (6) and (17). From a physical point of view, both expressions point to slightly different situations. When a thermal engine is tuned to supply work from a physical system out of equilibrium, its configuration depends on the state of the system. Each pure or mixed state requires different settings. Let $W_{i}$ be the work obtained from a system in a pure state $|r_{i}\rangle$. Next, we consider two cases:

(a) the pure state of each physical system is known, and the setting can be adjusted according to it. Then, the average work obtained by processing a collection of physical systems is the weighted average of all the $W_{i}$, each one contributing according to its corresponding eigenvalue $r_{i}$ in the density matrix $\rho^{⊗N}=\sum r_{i}|r_{i}\rangle \langle r_{i}|$. It is given by Equation (24).
(b) only the collective mixed state $\rho^{⊗NM}$ of the collection is known. Then, the engine is tuned with a different set of parameters, and the average value of the extracted work is lower than in the previous case. It is given in Equation (19).

Situations (a) and (b) correspond to the tight and loose-labeling techniques, respectively. Work is immediately translated by information heat engines into reservoir bits. The conversion factor is given in Equation (20). The difference in the average value of work in (a) and (b) translates exactly into MS(ρ) bits. The same results can be extended to the case when σ,ρ do not commute, provided that coding is defined in a suitable basis.

## 5. Conclusions

In this paper, we have described two ways to label physical systems grouped in clusters. In both procedures, unitary transformations operate on a Hilbert space determined by the cluster and additional informational qubits. In the tight-labeling method, the label identifies pure states of the cluster, while, in the loose-labeling case, the label is chosen at random from a collection of clusters that share the same mixed quantum state. The costs of both procedures have been deduced in the asymptotic limit of infinite equal systems. The evaluation has been made counting the number of informational qubits in the pure $|0\rangle$ state in the final and the initial situations. They are related to the von Neumann and relative entropies S(ρ),S(ρ||σ), where ρ is the state of the physical systems and σ is the state relative to which the labeling is optimized. In both processes, no manipulation of the physical system is attempted. Its intervention only exhibits an observational character.

We have shown that the atomic cost of tight-labeling converges to the sum of the von Neumann and relative entropies $W_{1}=S\left(\rho \right)+S\left(\rho ||\sigma \right)$. Remarkably, this result does not depend on the coding basis. However, in the loose-labeling case, the atomic cost depends not only on ρ,σ, but also on the coding basis. It is bounded by the relative entropy S(ρ||σ), to which it can converge when a right basis is chosen, as explained in Section 3.

Assuming this choice of basis, we have shown that the costs of labeling, both in the tight and loose versions, correspond to what thermodynamical processing predicts by combining the models of (a) work extraction from physical systems out of equilibrium, and (b) information heat engines powered by pure state qubits. Through writing and erasing labels, we have presented a procedure to trade relative entropy for von Neumann entropy of the physical system just by informational manipulation.

## Figures and Tables

**Figure A1:**
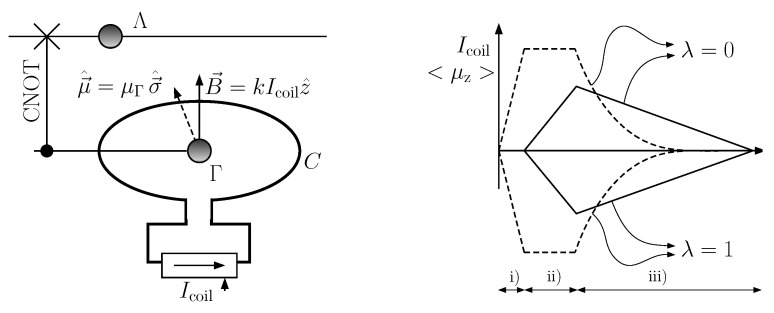
On the left, the basics components of the magnetic quantum information engine. The CNOT (Controlled NOT) gate is used to correlate the information Λ and the magnetic Γ qubits. The evolution of the electric current $I_{\mathrm{coil}}$ is controlled by the state of Λ. The magnetic induction field $\vec{B}$ at Γ that is generated by the coil is $kI_{\mathrm{coil}}\;\hat{z}$. The right part of the figure contains a graph of the evolutions od $I_{\mathrm{coil}},\;\langle \;\mu_{z}\;\rangle \;$ (solid and dashed lines, respectively). The upper branches represent the case λ=0, whereas the lower branches take place when λ=1, as described in Appendix A.

**Figure A2:**
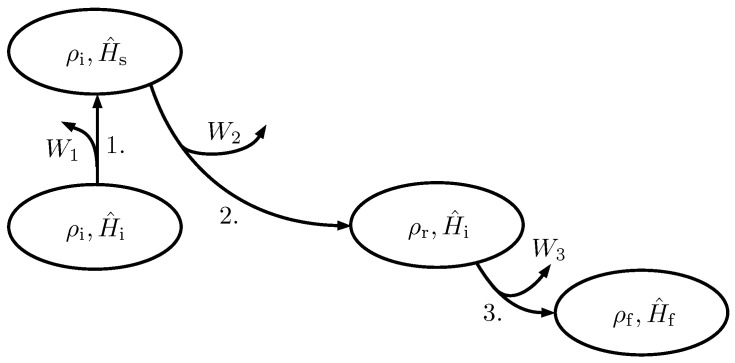
Representation of the process described in Appendix B for the work extraction in a process beginning with Hamiltonian ${\hat{H}}_{i}$ in state $\rho_{i}$ and ending in Hamiltonian ${\hat{H}}_{f}$ and state $\rho_{f}$. Stages 2 and 3 are isothermal.

## Appendix A. Magnetic Spin Information Heat Engine

In order to illustrate the connection between information reservoirs and thermal machines that is mentioned in the Introduction, this appendix describes a short version of a magnetic Quantum Information Heat Engine [17]. Its input is a low entropy qubit Λ from an information reservoir, and its output is twofold: the same qubit in a higher entropy state and electrical work is delivered to a magnetic coil through an induced electromotive force. The energy stems from a thermal reservoir at temperature *T*. The engine is reversible and can be employed to lower the entropy of qubits from an information reservoir if some work is supplied.

The physical system within the engine (see Figure A1) consists of an internal magnetic spin $\frac{1}{2}$ particle Γ that sits in the magnetic field generated by a classical induction coil *C*. The *z*-component of the magnetic moment of Γ is $\mu_{\Gamma }{\hat{\sigma }}_{z}$, where $\sigma_{z}$ is the third Pauli matrix. The machine operates cyclically, according to the following steps:

(i) The electric current in the coil $I_{\mathrm{coil}}$ is initially null. The qubit from the information reservoir enters in a partially mixed state $\lambda_{0}=q_{0}|0\rangle \langle 0|+\left(1-q_{0}\right)|1\rangle \langle 1|$. The magnetic qubit is initially in a maximally mixed state $\gamma_{0}=\frac{1}{2}\left(|↑\rangle \langle ↑|+|↓\rangle \langle ↓|\right)$. They undergo a CNOT (Controlled NOT) operation, controlled by the magnetic qubit. The result is an entangled mixed state:
  (A1) $$\rho_{\gamma \lambda }=\frac{q_{0}}{2}\left(|0↑\rangle \langle 0↑|+|1↓\rangle \langle 1↓|\right)+\frac{1-q_{0}}{2}\left(|0↓\rangle \langle 0↓|+|1↑\rangle \langle 1↑|\right),$$
  which defines two conditional states for the magnetic qubit Γ, each one corresponding to a state of the informational qubit Λ.
  (A2) $$\left\{\begin{array}{ccc} \lambda =|0\rangle \langle 0| & -> & \gamma_{\lambda =0}=q_{0}|↑\rangle \langle ↑|+\left(1-q_{0}\right)|↓\rangle \langle ↓|,\\ \lambda =|1\rangle \langle 1| & -> & \gamma_{\lambda =1}=q_{0}|↓\rangle \langle ↓|+\left(1-q_{0}\right)|↑\rangle \langle ↑|. \end{array}\right.$$
(ii) In this stage, the magnetic qubit Γ is isolated from the thermal reservoir. The current in the coil is raised to a value $I_{\mathrm{coil}}^{\left(\lambda =0,1\right)}$ controlled by λ. It is determined by making $\gamma_{\lambda =0,1}$ equal to the Gibbs state for the corresponding value of the magnetic field ${\vec{B}}_{\Gamma }=\;k\;I_{\mathrm{coil}}\;\hat{z}$, at Γ generated by the current $I_{\mathrm{coil}}$. The equation that fixes the two possible values of $I_{\mathrm{coil}}^{\left(\lambda =0,1\right)}$ is
  (A3) $$\gamma_{\lambda =0,1}=\frac{e^{\mu_{\Gamma }\;k\;I_{\mathrm{coil}}^{\left(\lambda =0,1\right)}/k_{B}\;T}|↑\rangle \langle ↑|+e^{-\mu_{\Gamma }\;k\;I_{\mathrm{coil}}^{\left(\lambda =0,1\right)}/k_{B}\;T}|↓\rangle \langle ↓|}{e^{\mu_{\Gamma }\;k\;I_{\mathrm{coil}}^{\left(\lambda =0,1\right)}/k_{B}\;T}+e^{-\mu_{\Gamma }\;k\;I_{\mathrm{coil}}^{\left(\lambda =0,1\right)}/k_{B}\;T}},$$
  which, together with Equation (A2), yields
  (A4) $$\left\{\begin{array}{ccc} I_{\mathrm{coil}}^{\left(\lambda =0\right)} & = & \frac{k_{B}\;T}{2\mu_{\Gamma }k}\ln\frac{q_{0}}{1-q_{0}},\\ I_{\mathrm{coil}}^{\left(\lambda =1\right)} & = & \frac{k_{B}\;T}{2\mu_{\Gamma }k}\ln\frac{1-q_{0}}{q_{0}}. \end{array}\right.$$
(iii) The current is gradually turned down until it is completely switched off. The process occurs slowly enough for assuming a thermal equilibrium state for Γ throughout this stage. Therefore, the final states for Λ,Γ are maximally mixed. Only the Λ qubit exits in a different state than it started in. The current and the Γ qubit are reset to their initial conditions.

Let $\Phi_{C->C},\Phi_{\Gamma ->C}$ be the contributions to the magnetic flux through the coil from its own current and the magnetic spin Γ, respectively. We further assume that the induction field ${\vec{B}}_{\Gamma }$ generated by the coil current $I_{\mathrm{coil}}$ at the position of Γ is $kI_{\mathrm{coil}}\;\hat{z}$. By the reciprocity theorem [46], we can state that $\Phi_{\Gamma ->C}=k\;\langle \;\mu_{z}\;\rangle \;$. The differential work on the coil can be evaluated by

(A5) $$dW_{\mathrm{coil}}=-I_{\mathrm{coil}}d\left(\Phi_{C->C}+\Phi_{\Gamma ->C}\right)=-I_{\mathrm{coil}}d\Phi_{C->C}-B_{\Gamma ,z}d\mu_{z},$$

which, in a cycle, integrates to

(A6) $$\oint dW_{\mathrm{coil}}=-\overset{=0}{\overset{︷}{\oint I_{\mathrm{coil}}d\Phi_{C->C}}}-\oint B_{\Gamma ,z}d\;\langle \;\mu_{z}\;\rangle \;=k\oint \;\langle \;\mu_{z}\;\rangle \;dI_{\mathrm{coil}},$$

where

(A7) $$\;\langle \;\mu_{z}\;\rangle \;=\mu_{\Gamma }Tr\;\left\{\gamma {\hat{\sigma }}_{z}\right\}=\mu_{\Gamma }\tanh\frac{\mu_{\Gamma }kI_{\mathrm{coil}}}{k_{B}\;T}.$$

The net energy supplied to the current source is

(A8) $$W^{\left(\lambda =0,1\right)}=k\oint \;\langle \;\mu_{z}\;\rangle {\;}^{\left(\lambda =0,1\right)}dI_{\mathrm{coil}}=kI_{\mathrm{coil}}^{\left(\gamma =0,1\right)}\;\langle \;\mu_{z}\;\rangle {\;}_{\mathrm{ii})}-k_{B}\;T\ln\underset{-\frac{1}{2}\ln\left(4q_{0}\left(1-q_{0}\right)\right)}{\underset{︸}{\cosh\frac{\mu_{\Gamma }kI_{\mathrm{coil}}^{\left(\gamma =0,1\right)}}{k_{B}\;T}}},$$

where $\;\langle \;\mu_{z}\;\rangle {\;}_{\mathrm{ii})}^{\left(\lambda =0,1\right)}$ is the value of $\;\langle \;\mu_{z}\;\rangle {\;}^{\left(\lambda =0,1\right)}$ throughout stage ii), which is easily evaluated to

(A9) $$\left\{\begin{array}{ccc} \;\langle \;\mu_{z}\;\rangle {\;}_{\mathrm{ii})}^{\left(\lambda =0\right)} & = & \mu_{\Gamma }\left(2q_{0}-1\right),\\ \;\langle \;\mu_{z}\;\rangle {\;}_{\mathrm{ii})}^{\left(\lambda =1\right)} & = & \mu_{\Gamma }\left(1-2q_{0}\right). \end{array}\right.$$

Now, Equation (A8) can be rewritten as

(A10) $$\left\{\begin{array}{ccc} W^{\left(\lambda =0\right)} & = & \frac{k_{B}\;T}{2}\left(\left(2q_{0}-1\right)\ln\frac{q_{0}}{1-q_{0}}+2\ln2+\ln q_{0}+\ln\left(1-q_{0}\right)\right),\\ W^{\left(\lambda =1\right)} & = & \frac{k_{B}\;T}{2}\left(\left(1-2q_{0}\right)\ln\frac{1-q_{0}}{q_{0}}+2\ln2+\ln q_{0}+\ln\left(1-q_{0}\right)\right), \end{array}\right.$$

which, in both cases λ=0,1, yields the same result:

(A11) $$W^{\left(\lambda =0,1\right)}=k_{B}\left(\ln2\right)T\left(1-S(\lambda_{0})\right),$$

which represents the potential of the qubits in the information reservoir to produce work. If qubits in a $\lambda_{0}=|0\rangle \langle 0|$ state are used, Equation (20) is obtained.

## Appendix B. Work Output from a System Out of Thermal Equilibrium

In this appendix, we derive the average work that can be obtained from a physical system in an initial state $\rho_{i}$ with Hamiltonian ${\hat{H}}_{i}$ when we reversibly drive the system to a thermal equilibrium state $\rho_{f}$ with Hamiltonian ${\hat{H}}_{f}$, related through Equation (18). The conclusion has already been given elsewhere [41], and we only present an alternative derivation that fits better with this paper. The process is reversible so that the work obtained is the maximum that can be achieved. We split the transformation into three steps (represented in Figure A2):

1. The Hamiltonian is changed from ${\hat{H}}_{i}$ to the operator ${\hat{H}}_{s}$ for which $\rho_{i}$ is in thermal equilibrium at temperature *T*. It is given by
  (A12) $${\hat{H}}_{s}=-k_{B}\left(\ln2\right)T\left(\log\rho_{i}+\log Z_{s}\right).$$
  In this step, the Hamiltonian varies quickly, so that the state does not change. Considering that there is no heat exchange in this step, the work obtained is
  (A13) $$W_{1}=\mathrm{Tr}\;\left\{\rho_{i}{\hat{H}}_{i}-\rho_{i}{\hat{H}}_{s}\right\}.$$
2. The Hamiltonian is slowly taken back to ${\hat{H}}_{i}$, while thermal equilibrium is assured. Therefore, the system is driven to the state $\rho_{r}$ given by:
  (A14) $$\rho_{r}=Z_{i}^{-1}\;e^{-\frac{{\hat{H}}_{i}}{k_{B}\;T}}.$$
  The second principle of Thermodynamics implies that the work extracted is
  (A15) $$W_{2}=k_{B}\left(\ln2\right)T\Delta S-\Delta U=k_{B}\left(\ln2\right)T\;\left(S\left(\rho_{r}\right)-S\left(\rho_{i}\right)\right)-\left(U\left(\rho_{r},{\hat{H}}_{i}\right)-U\left(\rho_{i},{\hat{H}}_{s}\right)\right),$$
  where $S\left(\rho \right)=-\mathrm{Tr}\;\left\{\rho \log\rho \right\}$ and $U\left(\rho ,\hat{H}\right)=\mathrm{Tr}\;\left\{\rho \hat{H}\right\}$. Taking into account Equations (A12) and (A14), $W_{2}$ evaluates to
  (A16) $$W_{2}=k_{B}\left(\ln2\right)T\left(\log Z_{i}-\log Z_{s}\right),$$
  which, together with $W_{1}$, adds up to
  (A17) $$W=W_{1}+W_{2}=k_{B}\left(\ln2\right)T\left(\mathrm{Tr}\;\left\{\rho_{i}\log\rho_{i}\right\}-\mathrm{Tr}\;\left\{\rho_{i}\log\rho_{r}\right\}\right)=k_{B}\left(\ln2\right)T\;S(\rho_{i}\left|\right|\rho_{r}).$$
3. While keeping thermal equilibrium, the Hamiltonian is taken from ${\hat{H}}_{i}$ to ${\hat{H}}_{f}$. The work is obtained as in the previous step and yields:
  (A18) $$W_{3}=k_{B}\left(\ln2\right)T\left(\log Z_{f}-\log Z_{i}\right).$$

The final count gives

(A19) $$W=W_{1}+W_{2}+W_{3}=k_{B}\left(\ln2\right)T\;S(\rho_{i}\left|\right|\rho_{r})+k_{B}\left(\ln2\right)T\left(\log Z_{f}-\log Z_{i}\right).$$

The quantity $F\left(\hat{H},T\right):=-k_{B}\left(\ln2\right)T\;\log Z\left(\hat{H},T\right)$ is known as the *free energy* for a physical system with Hamiltonian $\hat{H}$ in equilibrium at temperature *T*. Equation (A19) can be rewritten as

(A20) $$W=k_{B}\left(\ln2\right)T\;S(\rho_{i}\left|\right|\rho_{r})+F\left({\hat{H}}_{i},T\right)-F\left({\hat{H}}_{f},T\right).$$

In the context of this paper, we assume that ${\hat{H}}_{i}={\hat{H}}_{f}$ and the work that can be extracted from a system out of equilibrium is directly related to the relative entropy

(A21) $$W=k_{B}\left(\ln2\right)T\;S(\rho_{i}\left|\right|\rho_{f}).$$

## Appendix C. Label Trimming

In this appendix, we describe the trimming operation depicted in Figure 4. The w(c(ℓ)) leading qubits of label *L* hold the codeword assigned to the cluster. The trailing qubits are in a $|0\rangle$ state. The purpose of the operation is to replace them with worthless qubits in a maximally mixed state. It is split into two unitary operations. The first one performs a transformation $U_{\mathrm{trimming},1}$ on three labels $L,L^{\prime },D$. $L^{\prime }$ enters the process with all its tagging qubits in a $|0\rangle$ state, while those of *D* are maximally mixed. The operation is controlled by the qubits of *L* and copies its leading w(c(ℓ)) qubits to $L^{\prime }$. The remaining H−w(c(ℓ)) ones are copied from *D*. Now, $L^{\prime }$ holds the desired label. The following operation, carried out by the gate $U_{\mathrm{trimming},2}$, aims at resetting the *L* label and the trailing H−w(c(ℓ)) qubits of *D*. It is controlled by $L^{\prime }$ where the codeword is now stored. Considering the whole operation, H−w(c(ℓ)) valuable qubits are recovered in the process.

## Appendix D. Label Shuffling

In this appendix, we explain how the shuffling of labels depicted in Figure 5 can be implemented by a unitary transformation. We have split the process into three operations, performed by the unitary gates $U_{\mathrm{shuffling}},U_{\mathrm{which}\;p}$ and $U_{\mathrm{dispose}\;w}$. The first gate operates on three groups of tagging qubits: W,V are collections of *M* labels and *J* is a collection of *P* qubits, where *P* is equal to M!. Initially, *W* contains the labels generated for a group *E* of *M* clusters; all the tagging qubits in *V* are in a $|0\rangle$ state and all the qubits in *J* are in a maximally mixed state. The gate writes in *V* the values in *W* according to the *p*-th permutation of the labels $w_{1},\ldots ,w_{M}$. The value of *p* is read from the random group *J*. The unnecessary trailing qubits of each label are then trimmed according to the procedure described in Appendix C.

The second gate, $U_{\mathrm{which}\;p}$, identifies the *p* of the permutation from the values of W,V and resets the qubits of *J* that host the value of *p*.

The number of possible values for each label $w_{i}$ is $2^{N}$. Let $L_{i}$ be the *i*-th one and $n_{i}\left(F\right)$ the number of labels in the collection *F* that are equal to $L_{i}$. The number of permutations of the labels is

(A22) P(F)=Mn1(F),…,n2N(F).

The leading R(F):=⌊logP(F)⌋ qubits of $p_{1},\ldots ,p_{P}$ are reset to state $|0\rangle$ after the gate. For large *M*, $\frac{R(F)}{M}\to S_{L}$, where $S_{L}$ is the entropy associated with the probability distribution for the $2^{N}$ possible labels of the $2^{N}$ elements of the $B_{C}$ basis.

After the $U_{\mathrm{which}\;p}$ gate, *W* still holds the ordered collection of labels, while *V* holds a shuffled set. The last operation performed by $U_{\mathrm{dispose}w}$, resets the ordered set by using the collection of clusters $c_{1},\ldots ,c_{M}$ that generated them. The final state is a collection of reset labels at *W*, a shuffled set at *V*, and a collection of $|0\rangle$ state qubits that correspond to the leading R(F):=⌊logP(F)⌋ members of *J*.

## Appendix E. Example

In order to illustrate the contents of this paper, we next present a simple example. Let us assume that the *atoms* are magnetic spin $\frac{1}{2}$ systems in the presence of a Hamiltonian

(A23) $$\hat{H}=-\mu B\;\left(|↑\rangle \langle ↑|-|↓\rangle \langle ↓|\right),$$

which determines a Gibbs state

(A24) $$\tau =\frac{e^{\mu B/k_{B}\;T}|↑\rangle \langle ↑|+e^{-\mu B/k_{B}\;T}|↓\rangle \langle ↓|}{e^{\mu B/k_{B}\;T}+e^{-\mu B/k_{B}\;T}}.$$

If we have a cluster of *N* spins in a pure state $\rho_{↑}:=|↑\rangle \langle ↑|$, we know, from Equation (A21), that we can obtain an average work of

(A25) $$W_{ex}=Nk_{B}\left(\ln2\right)TS(\rho_{↑}\;||\;\tau )=Nk_{B}\left(\ln2\right)T\log\left(1+e^{-2\mu B/k_{B}\;T}\right).$$

The same value is necessary for the reverse operation, whereby a system of *N* spins in state $\rho_{↑}$ is obtained from *N* systems in state τ, provided that work $W_{ex}$ is supplied.

The same work can be obtained at an Information Heat Engine if $W_{ex}/k_{B}\left(\ln2\right)T$ bits from an information reservoir are used. This consideration sets an exchange rate between spins and bits given by $S(\rho_{↑}\;||\;\tau )$ bits per spin. We have described a particular model of Information Heat Engine that employs magnetic spins in [47]. Work is extracted by exposing the spins to a sequence of interactions with a suitable magnetic field in the presence of a heat bath at temperature *T*. The magnetic field is chosen according to the state of the spin.

Tight-labeling a cluster *C* of *N* spins implies applying a Shannon coding procedure that assigns a sequence *L* of tagging qubits to *C*. The coding procedure defined in Section 2 consists of two steps. We have found that the best way to keep track of the costs is to employ unitary transformations. They are defined by their action on the elements of a particular basis $B_{C}$. We require τ to be diagonal in $B_{C}$. For N>1, there are many possible choices of $B_{C}$. Let p(b) be the eigenvalue of τ for the element *b* of $B_{C}$. Our coding procedure begins by assigning to *b* a sequence of tagging qubits. The leading ⌈−logp(b)⌉ represents the Shannon code for *b*, while the trailing ones are $|0\rangle$ state qubits (see Figure 3). The operation is defined in Equation (2). Then, the valuable trailing qubits are recovered by the trimming procedure described in Appendix C and represented in Figure 4.

In Section 2, we prove that the tight-labeling of a cluster of *N* spins in state $\rho_{↑}$, according to a Shannon coding system optimized for a distribution of states given by τ, requires exactly $S\left(\rho_{↑}\right)+S(\rho_{↑}\;||\;\tau )=S(\rho_{↑}\;||\;\tau )$ bits per spin in the N→∞ limit. This is the same number of bits per spin found for the Thermodynamic approach at the beginning of this section. However, we would like to underline the different contexts in which they have been derived. They come as answers to the following questions:

1. How many bits from an information reservoir are needed to match the work that is required to take a cluster of *N* spins out of thermal equilibrium?
2. How many bits are needed to label the state of a cluster of *N* spins using a coding system optimized for a given distribution of states?

Note that, in the second context, there are no concepts like temperature, heat, work or Hamiltonian. However, the coincidence of the answers to both questions could have been predicted after some abstraction was made about the first situation. Considering the initial and final states of spins and information reservoir bits, the net result is the obtention of some spins in a known state after employing a number of bits from an information reservoir. In the labeling scenario, the same process has happened. Before the operation, in both cases, picking a cluster at random implies a quantum state τ for it. At the end, we can know that it is in state $\rho_{↑}$.

For the reverse process, given the unitarity of the tight-labeling procedure, un-labeling a state restores the $|0\rangle$ state of the label qubits. In the Thermodynamic scenario, processing a spin in state $\rho_{↑}$, produces an average amount of work that can be used to reset the given number of informational bits. Again, in both contexts, we see the same initial and final situations.

Collections of spin clusters sampled from the same state can then undergo a loose-labeling procedure by which their tight-labels are reassigned at random. In this particular example, defined by a pure state $\rho_{↑}$, all the labels are equal and no informational qubits are recovered. In fact, $S(\rho_{↑})=0$, so that the unitary costs of tight- and loose-labeling are the same and are given by the relative entropy $S(\rho_{↑}||\tau )$.

The most relevant result of the paper is that this situation holds even when the state to be labeled does not commute with the state σ that describes the underlying distribution, provided that a particular basis is chosen to define the coding procedure. In the example presented here, it is the basis $B_{M}$ that diagonalizes the magnitude and the *z* component of the total spin of the cluster.

Let us consider a state $|\to \rangle :=2^{-1/2}\left(|↑\rangle +|↓\rangle \right)$ and its density matrix $\rho_{\to }:=|\to \rangle \langle \to |$. Unlike $\rho_{↑}$, $\rho_{\to }$ does not commute with τ. It is impossible to find a basis that simultaneously diagonalizes $\rho_{\to }^{⊗N}$ and $\tau^{⊗N}$. Therefore, the tight-label assigned to ${|\to \rangle }^{⊗N}$ must be a linear combination of the labels defined for the elements of $B_{C}$. In the $B_{C}^{⊗M}$ basis, the collection of *M* systems in the $\sigma_{\to }$ state is described by a superposition of basis states. Some of them contain the same labels for the *M* clusters, but others do not and thus may undergo a shuffling process that allows the recovery of some reset qubits. Its average number per cluster is given by $S_{L}$, as defined in Appendix D. However, if the $B_{M}$ basis is chosen, then $S_{L}/N$ tends to 0 and the relative entropy is recovered as the unit cost, as explained in Section 3.

The same result extends to the case when the labels are shuffled in a collection of clusters at any mixed state ρ, whether or not it commutes with σ, as described in Section 3.
